# Early complications and quality of life in patients with immediately loaded implant-supported maxillary partial rehabilitations: A prospective cohort study

**DOI:** 10.4317/medoral.26158

**Published:** 2023-07-10

**Authors:** Alba Sánchez-Torres, Marta Moragón-Rodríguez, Alazne Agirre-Vitores, Iñaki Cercadillo-Ibarguren, Rui Figueiredo, Eduard Valmaseda-Castellón

**Affiliations:** 1 DDS, MS, Master of Oral Surgery and Implantology. Associate Professor of Oral Surgery and Professor of the Master’s degree program in Oral Surgery and Implantology, School of Medicine and Health Sciences, University of Barcelona. Researcher at the IDIBELL Institute. Spain; 2 DDS, MS. Master of Oral Surgery and Implantology. School of Medicine and Health Sciences, University of Barcelona. Spain; 3 DDS. Student of the Master of Oral Surgery and Implantology degree program, School of Medicine and Health Sciences, University of Barcelona. Spain; 4 DDS, MS, PhD, Master of Oral Surgery and Implantology. Professor of the Master’s degree program in Oral Surgery and Implantology, School of Medicine and Health Sciences, University of Barcelona. Researcher at the IDIBELL Institute. Spain; 5 DDS, MS, PhD, Master of Oral Surgery and Implantology. Associate Professor of Oral Surgery and Professor of the Master’s degree program in Oral Surgery and Implantology, School of Medicine and Health Sciences, University of Barcelona. Researcher at the IDIBELL Institute. Spain; 6 DDS, MS, PhD, EBOS. Professor of Oral Surgery and Director of the Master’s degree program in Oral Surgery and Implantology, School of Medicine and Health Sciences, University of Barcelona. Researcher at the IDIBELL Institute. Spain

## Abstract

**Background:**

Immediate loading of dental implants is considered an excellent option to reestablish function and aesthetics in a short period of time, thereby reducing the psychological impact of edentulism. The aim of this study was to describe the incidence of complications in immediately loaded implant-supported single or partial maxillary provisional rehabilitations; to assess changes in patient quality of life (QoL); to evaluate patient overall satisfaction; and to determine whether the occurrence of complications affects these outcomes.

**Material and Methods:**

Patients requiring partial rehabilitation with implants in the maxilla were included in a prospective cohort study. In all cases, implant-based restoration with an immediate loading protocol was indicated. A provisional restoration was placed within 72 hours after implant placement. Patient QoL was measured at the first appointment and just before placing the final restoration, using two validated questionnaires. All mechanical and biological complications occurring up until placement of the final restoration were documented. A descriptive and bivariate analysis of the data was performed.

**Results:**

Thirty-five patients with 40 prostheses supported by 60 implants were analyzed. Three implant failures were observed, yielding a 95% survival rate. Five provisional prosthesis fractures and two prosthetic screw loosenings were recorded in four patients. A significant reduction in OHIP-14 score was observed. Likewise, significant differences were found in the results of the QoLFAST-10, with a mean difference in score of 7.3 between the initial and final evaluation.

**Conclusions:**

Patients receiving immediately loaded implant-supported single or partial maxillary provisional rehabilitations seem to have a low risk of developing early mechanical (13.3%) or biological complications (5%). These patients appear to experience significant improvement in QoL and report excellent overall satisfaction with the treatment received - though the occurrence of complications seems to affect these outcomes.

** Key words:**Immediate loading, dental implants, implant failure, quality of life; mechanical complications.

## Introduction

Functional and aesthetic rehabilitation by means of dental implants has been widely described in the literature since the 1970s (1). The treatment concept, as well as the surgical and prosthetic protocols have evolved over the years, as reflected by different authors (2,3).

The placement of implants in fresh post-extraction sockets is a technically demanding procedure that decreases surgical morbidity and reduces the treatment time (4-7). These immediate post-extraction implants seem to have similar success rates in comparison with implants placed in fully healed alveolar ridges - thereby constituting a predicTable treatment option (8,9). Immediate loading is another option that reestablishes function and aesthetics in a short period of time, thereby reducing the psychological impact of edentulism (10). This leads to greater patient satisfaction, as well as to increased acceptance of implant treatment (3,11). Adequate primary stability of the implants is required. In this respect, a minimum insertion torque of 30 Ncm is recommended in placing the provisional prosthetic restoration, since without adequate stability, the osseointegration process might be compromised (10,12). Even though these treatments have shown high success rates (13-15), some complications may occur. Specifically, a number of studies have reported mechanical complications related to the immediately loaded provisional prostheses (16). Such problems seem to be more frequent in patients with risk factors (17). On the other hand, surgical site infections or implant failures (i.e., biological complications) may also be observed (18,19). Nevertheless, the overall outcomes of these treatments seem to be good even in full arch restorations, with reported patient-based success rates of > 80% after a mean follow-up of > 4 years (16).

Most studies employ clinical and radiological variables to assess the treatment outcomes. Although these variables are useful and objective, patient perceptions should also be taken into account. In recent years, some reports have assessed the treatment outcomes based on the degree of patient satisfaction from the aesthetic and functional perspectives (20).

To the best of our knowledge, no studies have specifically assessed early complications (i.e. complications that occur before the placement of the final restoration) in patients with immediately loaded implant-supported partial maxillary rehabilitations, taking patient opinion into account. Thus, the main aim of the present study was to describe the incidence of complications appearing before placement of the final restoration in immediately loaded implant-supported single or partial maxillary rehabilitations. The secondary aims were to assess the changes in patient quality of life; evaluate patient overall satisfaction; and determine whether the occurrence of early complications affects these outcomes.

## Material and Methods

- Study design

An observational prospective cohort study was carried out taking into consideration the recommendations of the STROBE statement (Strengthening the Reporting of Observational studies in Epidemiology) (21). The protocol was approved by the Bioethics Committee of the University of Barcelona (Ref. IRB00003099), and the study abided with the principles of the Declaration of Helsinki. Written informed consent was obtained from all the patients.

- Patient selection

The patients were recruited at the Dental Hospital of the University of Barcelona (Barcelona, Spain) from June 2017 until November 2020.

The inclusion criteria were: patients aged 18-80 years, requiring partial rehabilitation with implants in the maxilla, or with teeth that needed extraction. In all cases, implant restoration with an immediate loading protocol was indicated.

The exclusion criteria were: patients with uncontrolled systemic diseases (ASA score III or higher) that contraindicated surgical procedures or which could alter the healing process; uncontrolled periodontal disease or a plaque and/or bleeding on probing index score > 30%; heavy smokers (> 10 cigarettes/day); patients with severe attrition associated to bruxism; the presence of dehiscences and/or fenestrations during implant placement; patients with less than 8mm of residual ridge height or that needed vertical or horizontal bone augmentation procedures, and patients with relevant dental disease of the adjacent teeth.

- Sample size calculation

The sample size was calculated using the G* Power version 3.1.9.2 application (Universität Kiel, Germany). The primary outcome variable was the difference in overall Oral Health Impact Profile-14 (OHIP-14) score as recorded before and after treatment. The values reported by Raes *et al*. (22) were taken into consideration. With an alpha value of 0.05 and a beta value of 0.2, a total sample size of 35 patients was established.

- Treatment protocol and surgical technique

The surgical procedures were carried out by residents of the Master’s degree program in Oral Surgery and Implantology of the University of Barcelona. Three previously calibrated researchers (AST, MMR, AAV) compiled all the data and performed the follow-up appointments.

After collecting all relevant medical data and performing the clinical examination, panoramic radiographs and cone-beam computed tomography (CBCT) scans were obtained in all the participants. The treatment protocol was explained, placing special emphasis on the surgical procedure and possible complications. When needed, patients underwent periodontal treatment before implant placement.

**Figure 1:**
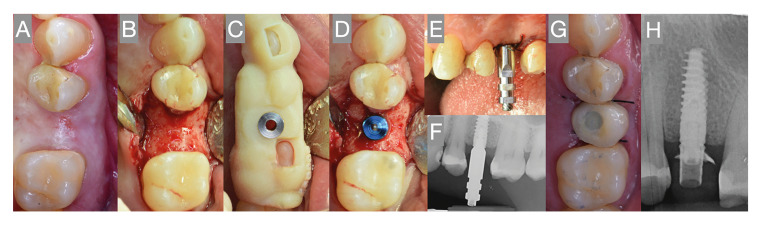
Main steps of the treatment protocol. A: Preoperative image; B: Flap elevation; C: Placement of the surgical guide; D: Drilling sequence and angulation pin; E: Impression transfer coping placement; F: Radiographic assessment before the impression; G: Provisional crown placement; H: Postoperative radiographic assessment.

The patients received 2 g of amoxicillin (or 600 mg of clindamycin in the case of allergy to amoxicillin) one hour before surgery, and local anesthesia was administered in the form of 4% articaine with epinephrine 1:100.000. In the case of immediate implant placement, atraumatic extractions were performed, and the socket walls were probed to confirm the absence of any anatomical defects or dehiscences. A mucoperiosteal flap was raised when needed, and implant drilling was performed following the instructions of the manufacturer. The implants (Ticare Inhex Quattro®, Mozo-Grau, S.A., Valladolid, Spain) were placed slightly subcrestal using a surgical splint to guarantee proper three-dimensional (3D) positioning. Non-absorbable sutures were employed to close the wound (Supramid 4/0, Aragó®, Barcelona, Spain). A minimum insertion torque of 35 Ncm was required to perform immediate dental impressions with an open-tray technique. A provisional restoration with ovoid pontics and without cantilevers was fitted by a clinician (AST) within 72 hours after implant placement. Fig. 1 shows the main steps of the treatment protocol. Occlusal adjustments were made to avoid strong contacts between the provisional and the opposing dentition. Sutures were generally removed 7 days after the surgical procedure, and the occlusal contacts were checked again. Periapical radiographs were obtained with the impression transfer coping and after placing the provisional crown. A delayed loading protocol was applied to implants with an insertion torque of < 35 Ncm.

The patients were informed about the most common postoperative complications, and were instructed to follow a soft diet during the provisional restoration stage.

The final restoration was placed 3-6 months after implant placement. Multiple restorations were splinted over transepithelial abutments. All structures were made using a CAD-CAM system (Ticare BioCam, Mozo-Grau, S.A., Valladolid, Spain). Patients were followed-up on until placement of the definitive prosthesis. At this appointment, the prostheses were removed to perform professional cleaning, and periapical radiographs were taken.

- Data collection

The following variables were recorded: age, gender, smoking habit, bruxism, history of periodontal disease, immediate or delayed implant placement, and follow-up time. Mechanical complications (screw or abutment loosening, prosthesis fracture) associated with the provisional prosthesis and early implant failures (i.e., failures occurring before the final restoration was placed) were also recorded.

To evaluate changes in patient quality of life (QoL) before and after treatment, validated Spanish versions of the Oral Health Impact Profile-14 (OHIP-14) (23) and Quality of life related to function, aesthetics, socialization, and thoughts about health-behavioral habits (QoLFAST-10) (24) questionnaires were completed by all participants at the first appointment and again just before definitive prosthesis placement. Moreover, the participants were asked to answer (“agree” / “neutral” / “disagree”) the following four questions about their overall satisfaction with the provided treatment: (a) Your prosthesis allows you to perform your daily oral hygiene correctly; (b) Your expectations were met; (c) You would repeat the treatment; (d) You would recommend the treatment to others.- 

- Statistical analysis

The data were processed using the Stata/IC 15.1 statistical package (StataCorp LLC, Lakeway Drive, USA). A descriptive and bivariate analysis was performed. A subgroup analysis was made to compare the changes in QoL between the patients that received immediate or delayed loading and the patients with or without complications, based on the Student t-test. Statistical significance was considered for *p*<0.05.

## Results

A total of 37 patients were enrolled in the study. Two patients were excluded on the basis of the inclusion and exclusion criteria. A total of 35 patients with 40 prostheses supported by 60 implants were thus finally analyzed. Thirty-one implants were placed in premolars, 11 in incisors, 9 in canines and 9 in first molars. All implants had a diameter of 3.75 or 4.25mm and the most common length was 13mm (*n*=30), followed by 11.5mm (*n*=16), 10mm (*n*=8), 15mm (*n*=4) and 8mm (*n*=2). Table 1 shows the main clinical features of the included patients. During the provisional period, three implant failures were recorded, yielding a 95% survival rate. Fourteen implants in 5 patients could not be immediately loaded due to a reduced primary stability. Four patients suffered mechanical complications during the follow-up period, as can be seen in Table 2 (5 provisional prosthesis fractures and 2 prosthetic screw loosenings). Fractures were solved by adding composite material at the same appointment.

**Figure 2:**
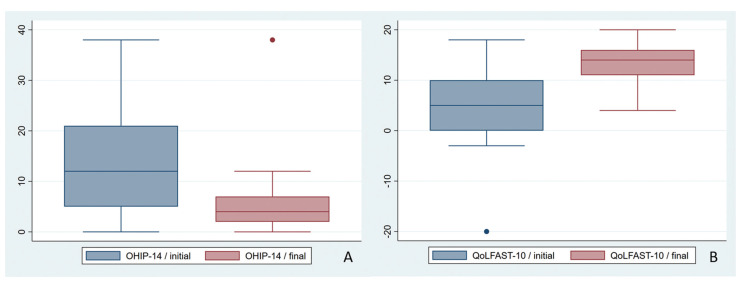
Box-plot showing the changes in the quality of life (OHIP-14 and QoLFAST scores) before and after treatment.

Overall, the patients significantly improved their quality of life as determined by the OHIP-14 (mean score reduction of 9.1) and QoLFAST-10 (mean score increase of 7.3) (Fig. 2, Table 3).

**Table 1:**
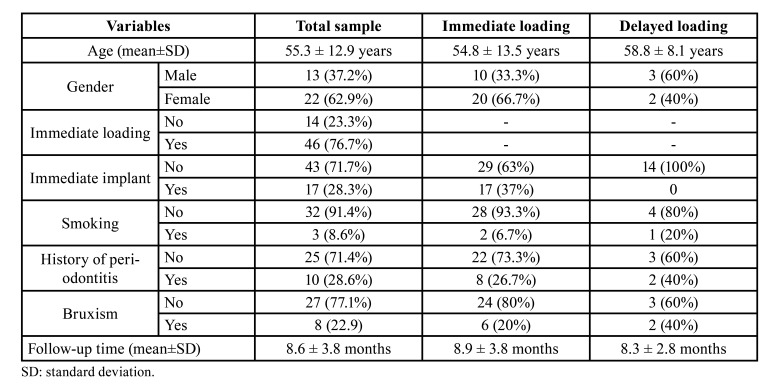
Main clinical features of the study sample. Data are also presented to allow comparison between the different loading protocols (immediate or delayed loading).

**Table 2:**
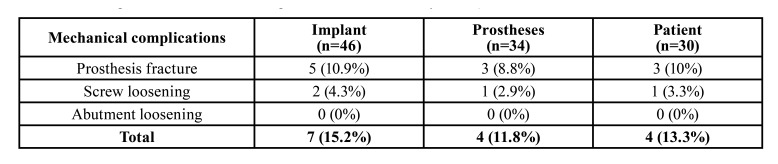
Mechanical complications associated to the immediately loaded provisional prosthesis (Note that in 3 patients that received several implants, at least one of the implants was not immediately loaded).

**Table 3:**
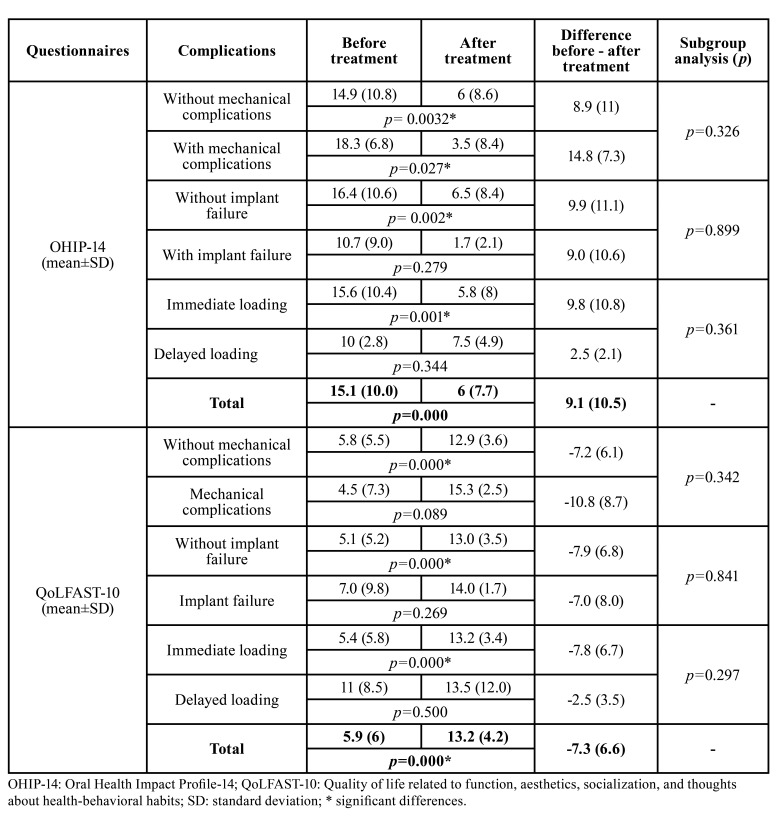
Quality of Life (QoL) and patient satisfaction.

Although no significant differences were found between the OHIP-14 and QoLFAST-10 scores of patients with and without mechanical or biological complications, significant improvement of the questionnaires scores was only observed in patients that had no implant failures (Table 3). Furthermore, the patients included in the immediate loading subgroup experienced significant improvement in QoL (as measured by the OHIP-14 and QoLFAST-10), while no significant differences were observed between the preoperative and postoperative questionnaire scores in the delayed loading subgroup (Table 3). All data regarding patient QoL can be observed in Table 3. The patient expectations were fulfilled in 95.6% of the cases, and all the participants felt that their prostheses allowed them to correctly clean the implants. All the included patients claimed that they would repeat and even recommend the treatment to others.

## Discussion

The results of the present study show that patients treated with immediately loaded implant-supported provisional prosthesis have a low incidence of mechanical and biological complications. Furthermore, the patients showed significant improvement of their QoL, except when mechanical or biological complications occurred.

De Rouck *et al*. (25) reported a 97% survival rate during the provisional period of single-tooth implant-supported restorations placed in the esthetic zone. Likewise, in a multicenter clinical study with a similar design that included 60 implants, a 98.3% cumulative survival rate was recorded (26). These values are slightly higher than that obtained in the present study (95%). The differences might be related to clinician experience, since all of our patients were treated by professionals with limited experience (< 3 years) in implant dentistry. Also, the present sample included posterior single-tooth restorations, which might be more susceptible to develop complications.

Some minor mechanical complications have been associated to immediately loaded implant-supported provisional prostheses (13,15). In the present study, 5 provisional prosthesis fractures and two prosthetic screw loosenings were recorded in four patients. Although the incidence of these events might be considered relevant (13.3%; Table 2), the clinical impact of such problems was small, since the great majority of them could be resolved in a single appointment and did not lead to major complications. Nonetheless, it is essential to warn patients that mechanical complications, if left untreated, might compromise the entire treatment. In fact, screw loosening or prosthesis fractures originate uncontrolled movements that can impair the osseointegration process. On the other hand, the present sample included patients with bruxism and with provisional prostheses placed in the upper arch, which have been considered to be risk factors for mechanical complications (16).

The use of patient-reported outcome measures (PROMs) to evaluate treatment modalities seems to be very useful, since clinical parameters might not reflect the patient expectations. This issue is especially relevant when comparisons are made between treatment options with similar clinical outcomes. Indeed, if the results reported by Benic *et al*. (10) are taken into account, there are no significant differences between immediate and conventional implant loading protocols in terms of implant survival rates and marginal bone levels. However, a recent systematic review has pointed out that patient satisfaction seems to be greater when immediate implant placement and immediate loading protocols are employed (27). Likewise, the results of the QoLFAST and OHIP-14 questionnaires obtained in the present study showed that when an immediate loading provisional prosthesis was placed, significant improvements in patient QoL were achieved. Thus, whenever possible, this option should be considered.

As mentioned above, a small proportion of patients experienced implant failures (3 patients) and mechanical complications (4 cases). These incidents seem to have an important impact upon patient QoL. Indeed, only patients that had an uneventful recovery until placement of the final restoration showed significant improvements in QoL (Table 3).

The placement of immediate post-extraction implants in periodontal infected sites is considered a controversial issue and many clinicians are reluctant to perform such treatments. Nevertheless, several authors have concluded that good results can be obtained in such situations, reducing the need to perform several surgical procedures (28,29). In the present sample, almost 30% of the patients had history of periodontitis and this variable was not associated with a higher risk of complications.

The outcomes of the present study should be interpreted with caution. Firstly, the sample size (35 patients) might be considered limited, especially considering the number of variables with confounding effects. However, the number of prostheses [40] and implants [60] analyzed was considered sufficient. Secondly, the present results can only be extrapolated to patients needing single of partial restorations in the upper arch - since the sample did not include full-arch prostheses or implants placed in the mandible. Another limitation of this research is related to the short follow-up period (up until placement of the final restoration), since it was focused on analyzing the complications and patient QoL associated to the provisional prosthesis. Therefore, future studies should be carried out to determine whether these clinical results and patient perceptions remain sTable over time. Finally, all treatments were performed by residents of a university master degree program with limited clinical experience (< 3 years); these results therefore probably would have improved if expert clinicians were involved.

## Conclusions

Taking into consideration the limitation of this study, immediately loaded implant-supported single or partial maxillary provisional rehabilitations seem to be a predicTable treatment option, with a low percentage of patients being affected by early mechanical (13.3%) or biological complications (5%). Furthermore, patients with immediately loaded restorations appear to significantly improve their quality of life and report high overall satisfaction with the treatment received. However, clinicians should be aware that the occurrence of complications during the provisional phase might affect these outcomes.
